# Thromboprophylaxis using combined intermittent pneumatic compression and pharmacologic prophylaxis versus pharmacologic prophylaxis alone in critically ill patients: study protocol for a randomized controlled trial

**DOI:** 10.1186/s13063-016-1520-0

**Published:** 2016-08-03

**Authors:** Yaseen M Arabi, Sami Alsolamy, Abdulaziz Al-Dawood, Awad Al-Omari, Fahad Al-Hameed, Karen E. A. Burns, Mohammed Almaani, Hani Lababidi, Ali Al Bshabshe, Sangeeta Mehta, Abdulsalam M. Al-Aithan, Yasser Mandourah, Ghaleb Mekhlafi, Simon Finfer, Sheryl Ann I. Abdukahil, Lara Y. Afesh, Maamoun Dbsawy, Musharaf Sadat

**Affiliations:** 1 Intensive Care Department, King Saud Bin Abdulaziz University for Health Sciences, King Abdullah International Medical Research Center, ICU 1425, PO Box 22490, Riyadh, 11426 Kingdom of Saudi Arabia; 2 Emergency Medicine and Intensive Care Department, King Saud Bin Abdulaziz University for Health Sciences, King Abdullah International Medical Research Center, Riyadh, Kingdom of Saudi Arabia; 3 Alfaisal University, Riyadh, Kingdom of Saudi Arabia; 4 Intensive Care Department, King Saud bin Abdulaziz University for Health Sciences, King Abdullah International Medical Research Center, Jeddah, Kingdom of Saudi Arabia; 5 Interdepartmental Division of Critical Care Medicine, Li Ka Shing Knowledge Institute, St Michael’s Hospital, Toronto, ON Canada; 6 Department of Pulmonary and Critical Care Medicine, King Fahad Medical City, ᅟRiyadh, ᅟSaudi Arabia; 7 King Saud Bin Abdulaziz University for Health Sciences, Riyadh, Kingdom of Saudi Arabia; 8 Department of Critical Care Medicine, King Khalid University, Assir Central Hospital, Abha, Kingdom of Saudi Arabia; 9 Medical/Surgical ICU, University of Toronto, Mount Sinai Hospital, Toronto, ON Canada; 10 Intensive Care Department, King Abdulaziz Hospital, Al Ahsa, Kingdom of Saudi Arabia; 11 Department of Intensive Care Services, Prince Sultan Military Medical City, Riyadh, Kingdom of Saudi Arabia; 12 International Extended Care Centers, ᅟJeddah, ᅟSaudi Arabia; 13 Intensive Care Royal North Shore Hospital of Sydney and Sydney Adventist Hospital, The George Institute for Global Health, Sydney Medical School, University of Sydney, Sydney, NSW Australia; 14 Intensive Care Department, King Abdulaziz Medical City, Riyadh, Kingdom of Saudi Arabia; 15 King Abdullah International Medical Research Center, Riyadh, Kingdom of Saudi Arabia

**Keywords:** Deep vein thrombosis, Pulmonary embolism, Intermittent pneumatic compression, Adjunct mechanical and pharmacologic DVT prophylaxis, Critically ill patients

## Abstract

**Background:**

Venous thromboembolism (VTE) remains a common problem in critically ill patients. Pharmacologic prophylaxis is currently the standard of care based on high-level evidence from randomized controlled trials. However, limited evidence exists regarding the effectiveness of intermittent pneumatic compression (IPC) devices. The Pneumatic compREssion for preventing VENous Thromboembolism (PREVENT trial) aims to determine whether the adjunct use of IPC with pharmacologic prophylaxis compared to pharmacologic prophylaxis alone in critically ill patients reduces the risk of VTE.

**Methods/Design:**

The PREVENT trial is a multicenter randomized controlled trial, which will recruit 2000 critically ill patients from over 20 hospitals in three countries. The primary outcome is the incidence of proximal lower extremity deep vein thrombosis (DVT) within 28 days after randomization. Radiologists interpreting the scans are blinded to intervention allocation, whereas the patients and caregivers are unblinded. The trial has 80 % power to detect a 3 % absolute risk reduction in proximal DVT from 7 to 4 %.

**Discussion:**

The first patient was enrolled in July 2014. As of May 2015, a total of 650 patients have been enrolled from 13 centers in Saudi Arabia, Canada and Australia. The first interim analysis is anticipated in July 2016. We expect to complete recruitment by 2018.

**Trial registration:**

Clinicaltrials.gov: NCT02040103 (registered on 3 November 2013).

Current controlled trials: ISRCTN44653506 (registered on 30 October 2013).

## Background

Venous thromboembolism (VTE), including both deep vein thrombosis (DVT) and pulmonary embolism (PE), is a common complication of critical illness and is associated with increased morbidity and mortality [1]. Supported by high-quality evidence, pharmacologic thromboprophylaxis is recommended for critically ill patients [2].

The evidence regarding the effectiveness of mechanical prophylaxis including an intermittent pneumatic compression device (IPC) and graduated compression stockings (GCS) for thromboprophylaxis is less clear. A systematic review by Limpus et al. included two randomized controlled trials that compared IPC to low-molecular-weight heparin (LMWH) in critically ill trauma patients [3, 4] and found no statistically significant difference in DVT rates between IPC and LMWH (risk ratio 2.37, 95 % 0.57–9.90) [5]. As such, clinical practice guidelines recommend that IPC be reserved for patients with contraindications to pharmacologic thromboprophylaxis [6]. In a cohort study using a propensity score-adjusted analysis, we found that incident VTE was lower with the use of IPC (versus no IPC) but not GCS (versus no GCS) [7]. This association of lower VTE with IPC was consistent regardless of the use and type of prophylactic heparin (unfractionated or LMWH), the type of admission (trauma or non-trauma, surgical or non-surgical). These findings suggest that IPC may provide thromboprophylaxis if used as an alternative to unfractionated heparin (UFH) or LMWH, and also when used as an adjunct to pharmacologic thromboprophylaxis. The largest trial to date on the effectiveness of IPC is the CLOTS 3 (Clots in Legs Or sTockings after Stroke) trial, which randomized 2876 stroke patients in 94 UK centers to IPC versus no IPC; the indication of pharmacologic prophylaxis was left to the appreciation of the treating team. The primary outcome was proximal vein DVT on screening ultrasound or any symptomatic DVT in the proximal veins, confirmed on imaging, within 30 days of randomization. The primary outcome occurred in 8.5 % of patients allocated to IPC and in 12.1 % of patients allocated to no IPC; with an absolute risk reduction of 3.6 % (95 % CI 1.4–5.8) [8]. Of note, fewer than 25 % of patients in CLOTS 3 received pharmacologic thromboprophylaxis. Nevertheless, the protective effect of IPC was observed whether pharmacologic thromboprophylaxis was or was not given. Regarding GCS, two trials (CLOTS 1 and CLOTS 2) documented a lack of effectiveness of GCS in thromboprophylaxis in stroke patients [9, 10].

The thromboprophylactic effect of IPC is thought to be related to enhancing venous blood flow in the lower extremities, increase in endogenous fibrinolysis, stimulation of vascular endothelial cells and a reduction in venous caliber [4]. In a study on normal volunteers the use of IPC was associated with increased endogenous fibrinolysis; tissue factor pathway inhibitor and plasminogen activator activity both increased after applying pneumatic compression for 2 h [11]. The IPC may have several hemodynamic effects; as it has been shown to augment venous return, increase central venous pressure and pulmonary arterial pressure [12] and increase cardiac output in healthy volunteers [13]. While the clinical implications of the hemodynamic effects of IPC remain unknown, one manufacturer (Tyco) lists cardiogenic pulmonary edema as a contraindication for IPC. Cutaneous complications are a concern with IPC; in CLOTS 3, lower extremity skin lesions were reported in 3 % of IPC patients versus 1 % of control patients (*p* = 0.002) [8].

The lack of clear evidence for IPC and GCS has been reflected in the wide variation in the use of these devices in surveys from Canada, France, Australia and Germany [5, 14–17], and more importantly, in the current practice guidelines. The American College of Physicians (ACP) guidelines for non-surgical patients recommend against the use of GCS, and suggest IPC as an alternative to pharmacologic thromboprophylaxis if the patient has a contraindication; but make no recommendation about its adjunct use to pharmacologic thromboprophylaxis [6]. In contrast, The American College of Chest Physicians’ (ACCP) 2012 guidelines recommend the use of GCS or IPC, although preference is given to IPC as an alternative but not as an adjunct to pharmacologic thromboprophylaxis in non-surgical critically ill patients [2].

### Study objectives

The primary objective of the PREVENT trial is to assess the superiority of adjunct use of IPC to pharmacologic thromboprophylaxis compared to pharmacologic thromboprophylaxis alone on the incidence of proximal DVT in critically ill patients.

### Secondary objectives

1. To study the effect of adjunct use of IPC on the incidence of pulmonary embolism and of distal lower extremity DVT
2. To study the effect of the adjunct use of IPC on intensive care unit (ICU), hospital and 90-day mortality and hospital length of stay (LOS)
3. To study the effect of adjunct use of IPC on hemodynamic status in terms of the need for vasopressor therapy
4. To study the effect of adjunct use of IPC on patients with heart failure in terms of ventilator-free days
5. To study the effect of adjunct use of IPC on VTE in the following subgroups: trauma, patients with central venous catheters in the femoral veins, stroke, postoperative patients, heart failure and shock
6. To examine whether there are differences in the effect of IPC based on whether UFH or LMWH is used
7. To examine whether there are differences in the thromboprophylactic effect between sequential and non-sequential IPCs
8. To examine whether there are differences in below-knee and above-knee sleeves
9. To examine whether IPC applied to the lower extremities reduces non-lower extremity thrombosis
10. To examine if there is dose-effect relationship of the IPC duration and incident DVT risk
11. To examine whether IPC increases the risk of skin ulcers or lower extremity ischemia and affects mobilization practice

### Tertiary objective

To test whether there are differences among different LMWHs (enoxaparin, dalteparin and others) in VTE prophylaxis compared to UFH.

## Methods/Design

### Design overview

The PREVENT trial is an international multicenter trial approved by the Institutional Review Board (IRB) of the Ministry of National Guard Health Affairs (MNGHA) in which eligible patients will be randomized to IPC or no IPC. The trial is registered in ClinicalTrials.gov: NCT02040103 and Current controlled trials: ISRCTN44653506. The study is sponsored by King Abdulaziz City for Science and Technology (AT 65 – 34) and King Abdullah International Medical Research Center (RC12/045/R), Riyadh, Saudi Arabia.

### Eligibility and enrollment

All patients admitted to the ICU will be screened for eligibility within the first 48 h of ICU admission. To enter the study, patients must fulfill the inclusion criteria and meet no exclusion criteria as detailed in Table 1. Eligible patients or their substitute decision-maker will be approached for written informed consent. No compensation is provided for enrollment in the trial.

**Table 1:** Study inclusion and exclusion criteria

Inclusion criteria • Medical-surgical ICU patients ≥14 years old at participating ICUs. ICUs that use other age cut-off for adult patients will adhere to their local standard (16 or 18 years) • Patient weight ≥45 kg • Expected ICU LOS ≥72 h • Eligible for pharmacologic thromboprophylaxis with UFH and LMWH	
Exclusion criteria • Patient treated with IPC for >24 h in this current ICU admission • Patient in the ICU for >48 h • Patient treated with pharmacologic VTE prophylaxis with medications other than UFH or LMWH • Inability or contraindication to applying IPC to both legs: ○ Burns in the lower extremities, lacerations, active skin infection and ischemic lower limb at the site of IPC placement ○ Acute ischemia in the lower extremities ○ Amputated foot or leg on one or both sides ○ Compartment syndrome ○ Severe peripheral arterial disease ○ Vein ligation, gangrene, recent vein grafts and draining incisions ○ Evidence of bone fracture in lower extremities • Therapeutic dose of anticoagulation with UFH or LMWH • Pregnancy • Limitation of life support, life expectancy ≤7 days or palliative care • Allergy to the sleeve material • Patients with inferior vena cava (IVC) filter	
Eligible non-randomized exclusion criteria ▪ Patient or substitute decision-maker declines consent ▪ Unable to obtain consent within 48 h of ICU admission ▪ ICU physician or other treating clinician declines consent ▪ Co-enrollment in trials with biologic interaction	

*ICU* intensive care unit, *IPC* intermittent pneumatic compression, *LMWH* low-molecular-weight heparin, *LOS* length of stay, *UFH* unfractionated heparin, *VTE* venous thromboembolism

### Informed consent

The study is conducted according to Good Clinical Practice guidelines. The study protocol as well as the informed consent have been approved by the IRB of King Abdullah International Medical Research Center, King Abdulaziz Medical City, Riyadh and the respective IRBs of all the other centers.

The research coordinator and/or physician investigator explains the objectives of the trial, and its potential risks and benefits, to the patient when possible or more commonly to his/her surrogate decision-maker. A witnessed written consent is obtained thereafter. The patient or surrogate decision-maker can withdraw from the study at any time without penalty or impact on patient care. A record is kept of all the patients who meet the inclusion criteria but are not randomized or withdrawn from the study.

### Trial interventions

The intervention group will receive IPC in addition to pharmacologic prophylaxis ordered by the treating team; the control group will receive pharmacologic prophylaxis only. All IPC devices intended for DVT prophylaxis can be used in the study. Sequential devices (with multi-chamber cuffs) are preferred, but non-sequential (with single-chamber cuffs) are acceptable. The type of device will be documented.

The use of IPC will follow the manufacturer’s instructions and local policies. IPC will be applied to both legs. We will use preferably thigh-length sleeves, but knee-length sleeves are acceptable. Foot pumps may be used in addition to the thigh- or knee-length sleeves. The ability to use IPC on both legs is one of the inclusion criteria. However, if during the study period the IPC could not be used on one leg, it should be continued on the other side and the use of a foot pump on the contraindicated leg considered if available. We aim to apply IPC continuously, both day and night for at least 18 h per day. The IPC may be removed during washing, physiotherapy, or screening compression duplex ultrasound. Nursing staff will record the application of IPC on the patient chart or on a daily data collection form designed to track adherence.

### Stopping guidelines for the intervention

Table 2 outlines the stopping guidelines for the intervention and the subsequent action. The intervention may be stopped in the following situations:

**Table 2:** Intervention stopping rules

Event	Action regarding the intervention	Action regarding other study procedures (data collection, ultrasounds, 28- and 90-day outcomes)
Suspected DVT	Hold intervention temporarily	Continue other study procedures
Confirmed DVT	Stop intervention permanently	This is a predefined endpoint
Suspected PE	Hold intervention temporarily	Continue other study procedures
Confirmed PE	Stop intervention permanently	This is a predefined endpoint
Non-lower extremity thrombosis	Continue intervention	Continue other study procedures
Anticoagulation for reasons other than PE or DVT	Continue intervention	Continue other study procedures
Prophylactic IVC filter placement	Continue intervention	Continue other study procedures
Severe skin ulcerations due to IPC	Hold intervention temporarily	Continue other study procedures
Ischemia due to IPC	Hold intervention temporarily	Continue other study procedures
Intolerance of IPC	Hold intervention temporarily	Continue other study procedures
Withdrawal from the study	Stop intervention permanently	Ask for permission to continue, if not stop.
Pharmacologic thromboprophylaxis is withheld	Apply IPC until pharmacologic prophylaxis is resumed (in both groups)	Continue other study procedures
Physician requests IPC in the control group	Place IPC and document the reason	Continue other study procedures
IPC not placed by mistake in the IPC group or placed by mistake in the control group	Resume the assigned intervention as soon as possible, document the reason, report as a violation	Continue other study procedures
Change in focus of care to palliation	Stop intervention permanently. IPC may be used at the discretion of the treating team	Stop ultrasounds. Continue other study procedures

*DVT* deep vein thrombosis, *IPC* intermittent pneumatic compression, *IVC* inferior vena cava, *PE* pulmonary embolus

1. Suspected or confirmed DVT: if there is suspicion of DVT, the IPC should be stopped until ultrasound excludes the diagnosis. If DVT is excluded, the IPC should be re-started
2. Suspected or confirmed PE: if there is suspicion of PE, the IPC should be stopped until the necessary work-up, such as spiral computed tomography (CT) scan, excludes the diagnosis. If PE is excluded, the IPC should be resumed
3. Pressure ulcer or ischemia that prevents the use of IPC
4. Change in focus of care to palliation
5. Patient is discharged from the ICU or has been in the trial for 28 days. At this point, the use of IPC is at the discretion of the treating team
6. Patient becomes fully mobile and no longer requires thromboprophylaxis in the judgment of their treating team

### Randomization

We will use a central, computer-based randomization system with variable blocks to conceal allocation. We will stratify based on center and pharmacologic thromboprophylaxis regimen (UFH or LMWH).

### Duration of the intervention

**Fig. 1:**
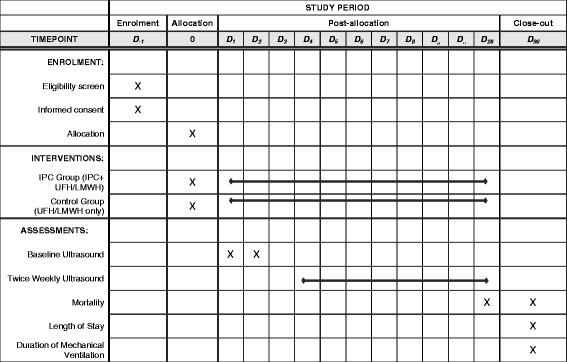
Schedule of enrollment, intervention and assessment for the thromboprophylaxis using combined intermittent pneumatic compression and pharmacologic prophylaxis versus pharmacologic prophylaxis alone in critically ill patients (PREVENT) trial

The study interventions will continue for the duration of the ICU stay or up to 28 days after randomization, after which the use of IPC will be at the discretion of treating team (Fig.1).

### Minimizing bias

#### Blinding

We aim to blind the radiologist interpreting the scans to detect DVTs and the study statistician. However, because of the nature of the intervention, the treating team and the ultrasonographer/technician performing the venous leg ultrasound will not be blinded to the intervention.

#### Minimizing contamination

Research coordinators will ensure enrollment of patients as quickly as possible after ICU admission. We will create a weekend on-call rota for the research coordinators in as many centers as possible. Patients receiving early IPC for more than 24 h will be excluded to eliminate early contamination.

#### Co-interventions

The ICU team will have full, independent control of patient management and as such, management other than IPC will not be influenced by the allocated intervention. The trial patients may have other risk factors that may modify the risk for development of DVT. Research coordinators will record all such risk factors. These may include drugs such as antiplatelet agents (aspirin, ticlopidine, clopidogrel) and anticoagulants that are started after randomization (warfarin, therapeutic-dose LMWH, therapeutic heparin administered intravenously, other anticoagulants). The use of these medications will be at the discretion of the clinical team. The use of GCS will not be permitted; if used the reason will be documented.

### Outcomes and follow-up

#### Primary outcome

The primary outcome is incident proximal lower extremity DVT detected after the third calendar day of enrollment. Certified ultrasonographers will perform routine twice weekly bilateral proximal lower extremity venous ultrasound, and if DVT is clinically suspected. The first ultrasound is performed within 48 h of enrollment. Prevalent DVT is defined as DVT documented within the first three calendar days of enrollment, and is considered to reflect a baseline characteristic and less likely be related to the study intervention. Patients with prevalent DVT will be included in the main analysis but a prevalent thrombosis is not considered a primary outcome.

We focus on proximal DVTs because they are much more reliably detected by ultrasound and are generally regarded as clinically more important. We will use the methodology used in the PROTECT trial to screen for proximal lower extremity DVTs [18]. The venous system will be examined at 1-cm intervals, documenting compressibility at the following six sites: common femoral, proximal superficial femoral, mid superficial femoral, distal superficial femoral, popliteal veins and trifurcation. DVT in any of these six sites qualifies as the primary outcome.

We will follow the same definitions used for VTE/DVT in the PROTECT trial [18]. We define DVT if there is a partially or completely incompressible venous segment. Venous wall thickening is not considered diagnostic of DVT. If a venous segment is not well visualized and is never well visualized on subsequent ultrasounds, the test is considered indeterminate; such events will be recorded but are not considered trial outcomes. Ultrasonographers may scan the distal leg veins at their discretion [18]. Distal DVT will be documented as a secondary endpoint.

DVTs are considered chronic if a test prior to enrollment reveals evidence of thrombus in the same or contiguous venous segment. DVTs and other VTE events are labeled as incident if they occur more than 3 calender days after randomization. We define a thrombus as catheter-related if a catheter had been in situ in the same or a contiguous venous segment within 3 calender days of the diagnosis. This definition is consistent with the definition used in the PROTECT protocol.

If clinicians suspect any VTE event, they will perform tests as clinically indicated.

#### Secondary outcomes

1. All incident DVTs including distal DVTs
2. All DVTs including incident and prevalent DVTs
3. Pulmonary embolism: we will follow the same definition used in the PROTECT trial. We define PE as: definite PE (characteristic intraluminal filling defect on thoracic CT scan, high-probability ventilation/perfusion (VQ) scan or detected at autopsy); probable PE (moderate-high pretest probability (high clinical suspicion) and no test or a non-diagnostic test); possible PE (low pre-test probability (low clinical suspicion and a non-diagnostic test); or no PE (negative or normal test without reference to pretest probability). PE will be followed up to ICU discharge or day 28 after randomization
4. Non-lower extremity non-PE thrombotic events
5. All VTE events
6. Skin pressure ulcers using the National Pressure Ulcer Advisory Panel (NPUAP) classification
7. Lower extremity ischemia
8. Daily level of mobility
9. Vasopressor requirements and vasopressor-free days (at day 28)
10. Duration of mechanical ventilation and mechanical ventilation-free days (at day 28)
11. ICU length of stay and ICU-free days (at day 28)
12. Serial cardiovascular and respiratory Sequential Organ Failure Assessment (SOFA) scores
13. Mortality:
  - ICU mortality: death in the ICU during the same ICU admission
  - Hospital mortality: death in the hospital (in the ICU or on the ward) during the same hospital admission. Hospital mortality will be censored at 1 year from the date of enrollment
  - 28-day mortality: death before or at day 28 of enrollment
  - 90-day mortality: death before or at day 90 of enrollment
14. Composite VTE events and 28-day mortality
15. Number of diagnostic tests for VTE including ultrasounds, spiral CT scans and VQ scans
16. Serious adverse events (Table 3)

**Table 3:** Deviations, violations and serious adverse events (SAEs)

	Conditions/events	Actions
Deviation	• Follow-up ultrasound not performed • Follow-up ultrasound performed late • Use of GCS for non-protocolized reasons	• Document in the CRF/eCRF • Prepare a note to file • Notify methods center
Violation	• Enrolling a non-eligible patient • Admission/baseline ultrasound not done within 48 h of enrollment • Received wrong intervention	• Prepare a note to file • Notify site PI • Notify IRB and sponsor immediately • Submit a written report within 7 days
Serious adverse event	• Skin ulceration categories III and IV • Ischemia due to IPC	• Notify site PI • Notify IRB and sponsor immediately

*CRF* case report form, *eCRF* electronic case report form, *IPC* intermittent pneumatic compression, *IRB* Institutional Review Board, *PI* physician investigator

### Data management

Data are entered through a password-protected access to an electronic database through an online portal and are stored on a secure server at King Abdullah International Medical Research Center, Riyadh, Saudi Arabia. The database includes multiple logic checks for double entry and range checks for data values. Several procedures to ensure data quality and protocol standardization are undertaken including: (1) training sessions for research coordinators from participating centers prior to study commencement, (2) a detailed Study Instruction Manual which outlines each step of the protocol, and (3) startup meetings for all sites, either by a physical conference or via videoconferencing. Patient personal data are de-identified. Each site investigator and coordinator have access to patients’ data from their site; the PI and the main coordinator has access to the data from all sites.

### Data analysis

#### Sample size calculation

Based on our previous observational study [7], the VTE risk (including DVT and PE) with no IPC was 7.2 % and with IPC it was 4.8 %. However, in this study no surveillance ultrasounds were performed. The PROTECT trial [13] documented a baseline risk for proximal DVT of 5.8 % in patients receiving unfractionated heparin and 5.1 % in patients receiving dalteparin. In addition, the PROTECT trial had a prevalent DVT rate at initial screening of 3.5 %. In the CLOTS 3 trial, the risk of DVT without IPC was 12.1 % and with IPC 8.5 %. We anticipate a higher baseline DVT rate than PROTECT because of the wider inclusion criteria and the inclusion of trauma patients; and lower than CLOTS 3 in which no pharmacologic prophylaxis was used routinely. Therefore, we anticipate a baseline risk of 7 % and absolute risk reduction of 3 %. Thus, a sample size of 1000 subjects in each group (accounting for a 5 % prevalent DVT rate and 5 % loss to follow-up) will have 80 % power to detect an absolute risk reduction of 3 %.

#### Statistical analysis

We will compare incident proximal lower extremity DVT between groups using the chi-square test. The unadjusted Cox proportional hazards model will be used to test the null hypothesis and will be used as a secondary analysis tool. A detailed statistical analysis plan will be published separately.

### Research governance

The study Steering Committee members will be responsible for overseeing the conduct of the trial, for upholding or modifying study procedures as needed, addressing challenges with protocol implementation, formulating the analysis plan, reviewing and interpreting the data and preparing the manuscript. This will be achieved through meetings (in-person or by conference calls) at least quarter-yearly. The study Data Safety and Monitoring Board (DSMB) will provide independent input regarding the safety and/or efficacy of the intervention. Upon completion, the results of the trial are planned to be published in a peer-reviewed journal. Authorship will follow the Uniform Requirements for Manuscripts Submitted to Biomedical Journals [19].

## Discussion

To our knowledge, this is the first RCT that compares adjunct IPC and pharmacologic prophylaxis versus pharmacologic prophylaxis alone in critically ill patients. To enhance the external validity of our findings, patients will be enrolled from more than 20 hospitals internationally. Central randomization with concealed allocation, blinded radiologist interpretations and adherence to the intention-to-treat principle will limit potential sources of bias. In addition, trial interventions and outcome monitoring will ensure that “loss to follow-up” for the primary outcome is minimal or absent.

The issue of the safety of critically ill patients is a prime concern in this randomized trial. Several measures have been taken to minimize, observe and document any potential safety concerns (Table 3).

The main limitation to our study is the inability to blind patients, their caregivers and the ultrasonographer/technician performing ultrasound with regard to allocation due to the nature of the intervention. We gave careful consideration to applying sham IPC in the control group. After discussion and deliberation, we decided not to include a sham IPC in the control group due to the potential harm of placing a cuff on the legs without inflation.

We used twice-weekly surveillance ultrasound to assess the primary outcome, rather than relying on DVT clinically suspected by clinicians, because the latter tends to underestimate the true incidence of DVT. We used a pragmatic time window of 48 h to perform the first (baseline ultrasound), and we considered the DVTs identified on this ultrasound as prevalent DVTs. Because we are interested in examining whether IPC reduces the incidence of DVT, our primary endpoint is DVTs that occur in patients who have a normal initial ultrasound but develop DVT as documented on subsequent exams; i.e., after the third calendar day (incident DVT).

Our primary endpoint is proximal DVT by surveillance ultrasound. We will not screen for distal DVTs because of the debated clinical relevance [20] and because the diagnosis by Doppler ultrasound is technically challenging and results may be inconsistent as reported by the CLOTS 3 trial [8]. However, we will document distal DVT if diagnosed by ultrasound obtained by the treating team.

We will also capture data on other outcomes relevant to IPC use including lower extremity ischemia, pressure ulcers and mobility. We will follow patients for development of safety concerns (lower extremity ischemia and pressure ulcers) as CLOTS 3 documented lower extremity skin lesions in 3 % of patients allocated to IPC and in 1 % of those allocated no IPC (*p* = 0.002) [8]. We will document the incidence of lower extremity ischemia, although there is no evidence that IPC causes ischemia. Additionally, based on clinical concerns that IPC use may negatively impact mobilization, we will evaluate whether IPC application reduces the chances of patients being mobilized.

We followed a pragmatic approach in selecting IPC devices and sleeve lengths, permitting treating teams at participating centers to use their own devices (sequential on non-sequential) and to select the sleeves (knee-length versus thigh-length) because at present there is no evidence for superiority of any of these choices over others.

The results of this study will contribute to a better understanding of the effectiveness of IPC in critically ill adults. In addition, the PREVENT trial will likely contribute to future clinical practice guidelines and patient safety initiatives by providing evidence that will inform practice regarding the best thromboprophylaxis for critically ill adult patients.

## Trial status

The first patient was enrolled in July 2014. As of May 2016, a total of 650 patients have been enrolled from 13 centers in Saudi Arabia, Canada and Australia. The first interim analysis is anticipated in July 2016. We expect to complete recruitment of 2000 patients by 2018.

## Abbreviations

ACCP, American College of Chest Physicians; DVT, deep vein thrombosis; GCS, graduated compression stockings; ICU, intensive care unit; IPC, intermittent pneumatic compression; LMWH, low-molecular-weight heparin; LOS, length of stay; PE, pulmonary embolism; RCT, randomized controlled trial; UFH, unfractionated heparin; VTE, venous thromboembolism
