# Erratum to: Thromboprophylaxis using combined intermittent pneumatic compression and pharmacologic prophylaxis versus pharmacologic prophylaxis alone in critically ill patients: study protocol for a randomized controlled trial

**DOI:** 10.1186/s13063-016-1556-1

**Published:** 2016-08-23

**Authors:** Yaseen M. Arabi, Sami Alsolamy, Abdulaziz Al-Dawood, Awad Al-Omari, Fahad Al-Hameed, Karen E. A. Burns, Mohammed Almaani, Hani Lababidi, Ali Al Bshabshe, Sangeeta Mehta, Abdulsalam M. Al-Aithan, Yasser Mandourah, Ghaleb Almekhlafi, Simon Finfer, Sheryl Ann I. Abdukahil, Lara Y. Afesh, Maamoun Dbsawy, Musharaf Sadat

**Affiliations:** 1 Intensive Care Department, King Saud Bin Abdulaziz University for Health Sciences, King Abdullah International Medical Research Center, ICU 1425, PO Box 22490, Riyadh, 11426 Kingdom of Saudi Arabia; 2 Emergency Medicine and Intensive Care Department, King Saud Bin Abdulaziz University for Health Sciences, King Abdullah International Medical Research Center, Riyadh, Kingdom of Saudi Arabia; 3 Alfaisal University, Riyadh, Kingdom of Saudi Arabia; 4 Interdepartmental Division of Critical Care Medicine, Li Ka Shing Knowledge Institute, St Michael’s Hospital, Toronto, ON Canada; 5 Department of Pulmonary and Critical Care Medicine, King Fahad Medical City, Riyadh, Saudi Arabia; 6 King Saud Bin Abdulaziz University for Health Sciences, Riyadh, Kingdom of Saudi Arabia; 7 Department of Critical Care Medicine, King Khalid University, Assir Central Hospital, Abha, Kingdom of Saudi Arabia; 8 Medical/Surgical ICU, University of Toronto, Mount Sinai Hospital, Toronto, ON Canada; 9 Intensive Care Department, King Abdulaziz Hospital, Al Ahsa, Kingdom of Saudi Arabia; 10 Department of Intensive Care Services, Prince Sultan Military Medical City, Riyadh, Kingdom of Saudi Arabia; 11 International Extended Care Centers, Jeddah, Saudi Arabia; 12 Intensive Care Royal North Shore Hospital of Sydney and Sydney Adventist Hospital, The George Institute for Global Health, Sydney Medical School, University of Sydney, Sydney, NSW Australia; 13 Intensive Care Department, King Abdulaziz Medical City, Riyadh, Kingdom of Saudi Arabia; 14 King Abdullah International Medical Research Center, Riyadh, Kingdom of Saudi Arabia

## Erratum

Unfortunately, the original version of this article [1] contained an error. An author’s family name was spelt incorrectly. Ghaleb Mekhlafi should be Ghaleb Almekhlafi. There will also be an update to make this correction.

## References

[1] Arabi YM, Alsolamy S, Al-Dawood A, Al-Omari A, Al-Hameed F, Burns KEA, Almaani M, Lababidi H, Al Bshabshe A, Mehta S, Al-Aithan AM, Mandourah Y, Almekhlafi G, Finfer S, Abdukahil SAI, Afesh LY, Dbsawy M, Sadat M (2016). Thromboprophylaxis using combined intermittent pneumatic compression and pharmacologic prophylaxis versus pharmacologic prophylaxis alone in critically ill patients: study protocol for a randomized controlled trial. Trials.

